# Recurrent intraventricular thrombus six months after ST-elevation myocardial infarction in a diabetic man: a case report

**DOI:** 10.1186/1756-0500-6-348

**Published:** 2013-09-02

**Authors:** Juan Lacalzada, Belén Marí, María Manuela Izquierdo, Alejandro Sánchez-Grande, Alejandro de la Rosa, Ignacio Laynez

**Affiliations:** 1 Department of Cardiology, Cardiac Imaging Laboratory, University Hospital of the Canary Islands, Ofra s/n. La Cuesta, La Laguna, Tenerife, 38320, Spain

**Keywords:** Acute myocardial infarction, Percutaneous coronary intervention, Drug-eluting stent, Echocardiography, Intraventricular thrombus, Dual antiplatelet therapy, Oral anticoagulation

## Abstract

**Background:**

Percutaneous coronary intervention with placement of a drug-eluting stent in a diabetic patient with ST-elevation myocardial infarction is a relatively common procedure, and always requires subsequent treatment with dual antiplatelet therapy. It is sometimes necessary to add oral anticoagulation therapy because of individual clinical circumstances, which further increases the risk of bleeding.

**Case presentation:**

A 66-year-old hypertensive diabetic man with a history of gastrointestinal bleeding was admitted with an ST-elevation inferior myocardial infarction that had been evolving over 72 h. Electrocardiography showed ST segment elevation in the inferior leads and Q waves in the inferior and anterior leads. He reported a similar episode of chest pain 1 month previously, for which he had not sought medical treatment. Coronary angiography showed chronic occlusion of the mid-left anterior descending coronary artery, and acute occlusion of the mid-right coronary artery. He was treated by percutaneous coronary intervention, with placement of a drug-eluting stent in the right coronary artery. Soon after admission, transthoracic echocardiography showed abnormal left ventricular contractility and a large left intraventricular thrombus. Three weeks after admission, the patient was discharged on dual antiplatelet therapy (clopidogrel and aspirin) and oral anticoagulation therapy (acenocoumarol). Four months after discharge, transthoracic echocardiography showed absence of left ventricular thrombus and resolution of the abnormal contractility in the area supplied by the revascularized right coronary artery. Given the high risk of bleeding, oral anticoagulation therapy was stopped. Six months later, transthoracic echocardiography showed recurrent left ventricular apical thrombus, and an underlying hypercoagulable state was ruled out. Oral anticoagulation therapy was restarted on an indefinite basis, and dual antiplatelet therapy was continued.

**Conclusions:**

The present case illustrates the need for repeat transthoracic echocardiography following the withdrawal of oral anticoagulation therapy in patients with ST-elevation myocardial infarction, both to monitor thrombus status and to assess left ventricular segmental contraction. In patients who require anticoagulation, avoidance of a drug-eluting stent is strongly preferred and second-generation stents are recommended. The alternative regimen of oral anticoagulation and clopidogrel may be considered in this scenario. In patients with recurrent intraventricular thrombus an underlying hypercoagulable state should be ruled out.

## Background

Use of drug-eluting stents in diabetic patients with ST-elevation myocardial infarction (STEMI) is relatively common, and subsequent treatment with dual antiplatelet therapy (DAPT) is invariably required [1]. Concomitant oral anticoagulation therapy (OAC) is sometimes necessary if the patient has left ventricular (LV) apical thrombus. Current guidelines on the management of patients with STEMI emphasize the role of transthoracic echocardiography (TTE) for assessment of the extent and degree of wall motion abnormalities and mural thrombi that would necessitate anticoagulation [2]. However, the lack of prospective randomized studies precludes the development of firm recommendations regarding the use of triple antithrombotic therapy (combined OAC and DAPT) in patients at high risk of bleeding. The European Heart Journal guidelines of 2012 [2] state that patients with mural thrombi require OAC with vitamin K antagonist therapy for up to 6 months. According to the 2013 American College of Cardiology/American Heart Association (ACC/AHA) guidelines [3], vitamin K antagonist therapy can be limited to 3 months in patients who have LV thrombus or are at risk for LV thrombus, such as patients with antero-apical akinesis or dyskinesis.

Triple antithrombotic therapy increases the risk of bleeding, and the optimal duration of triple therapy is unclear, especially in the era of stenting and DAPT. Decisions regarding administration of triple therapy after STEMI should consider stent placement, stent type, and the relative risks of bleeding and stent thrombosis [4,5].

If LV imaging after 3 months of therapy shows no evidence of thrombus, discontinuation of OAC earlier than 6 months can be considered, especially if there is recovery of apical wall motion [6]. Given the increased risk of bleeding related to dual and triple antithrombotic therapy, it would be desirable to test simpler drug combinations, and to clarify the optimal duration of treatment for prevention of further ischemic/thrombotic events.

We present here an elderly diabetic and hypertensive man who was admitted with acute inferior STEMI. He reported an episode of severe chest pain 1 month previously, for which he had not sought medical treatment. We discuss the unexpected developments and the resolution of his intraventricular thrombus, as well as potential emerging management strategies for such patients.

## Case presentation

**Figure 1:**
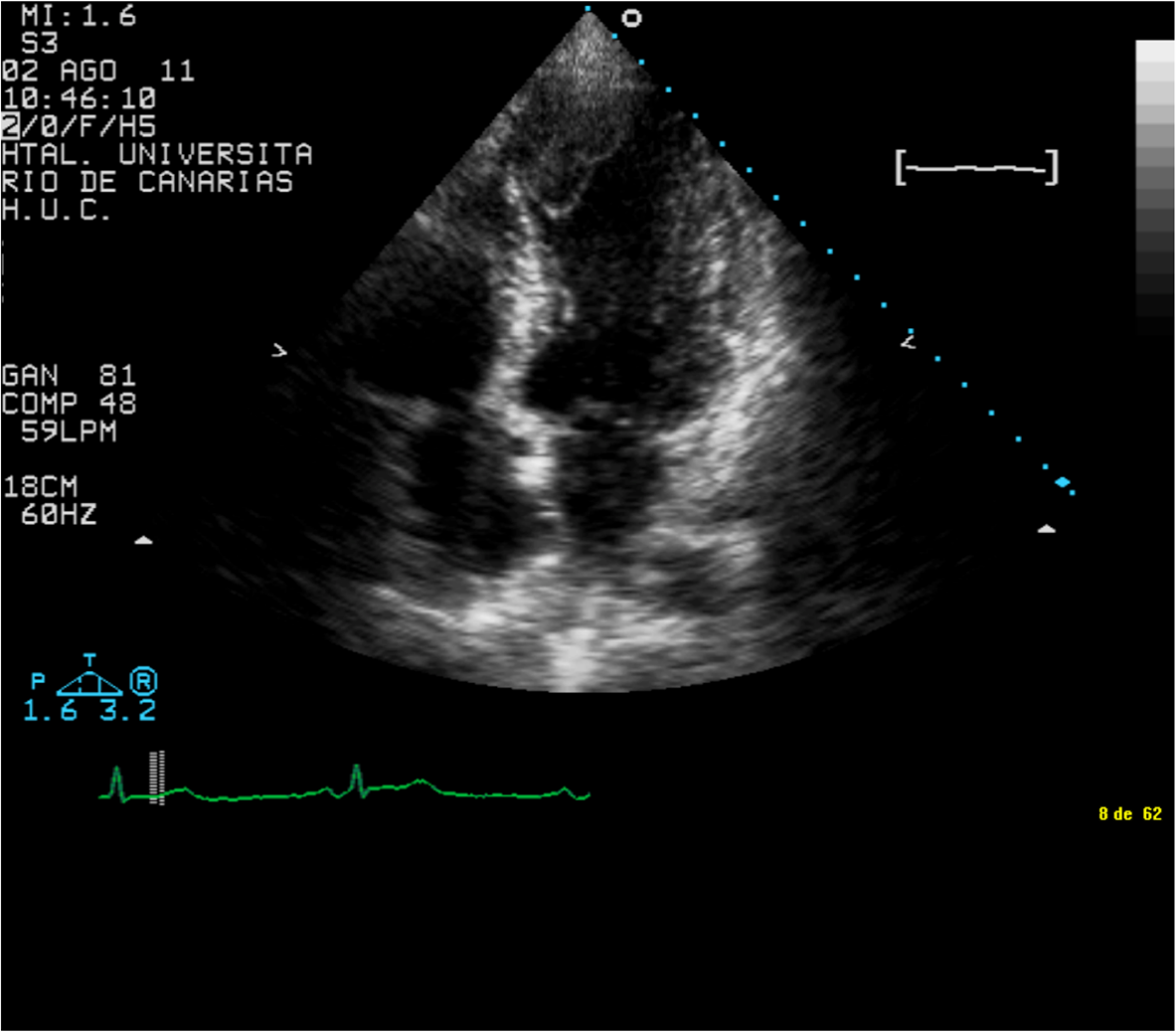
Early after admission, transthoracic echocardiography showed left ventricular inferior hypokinesis, anterior and apical akinesis, and a large intraventricular thrombus adherent to the septoapical and infero-apical endocardium.

**Figure 2:**
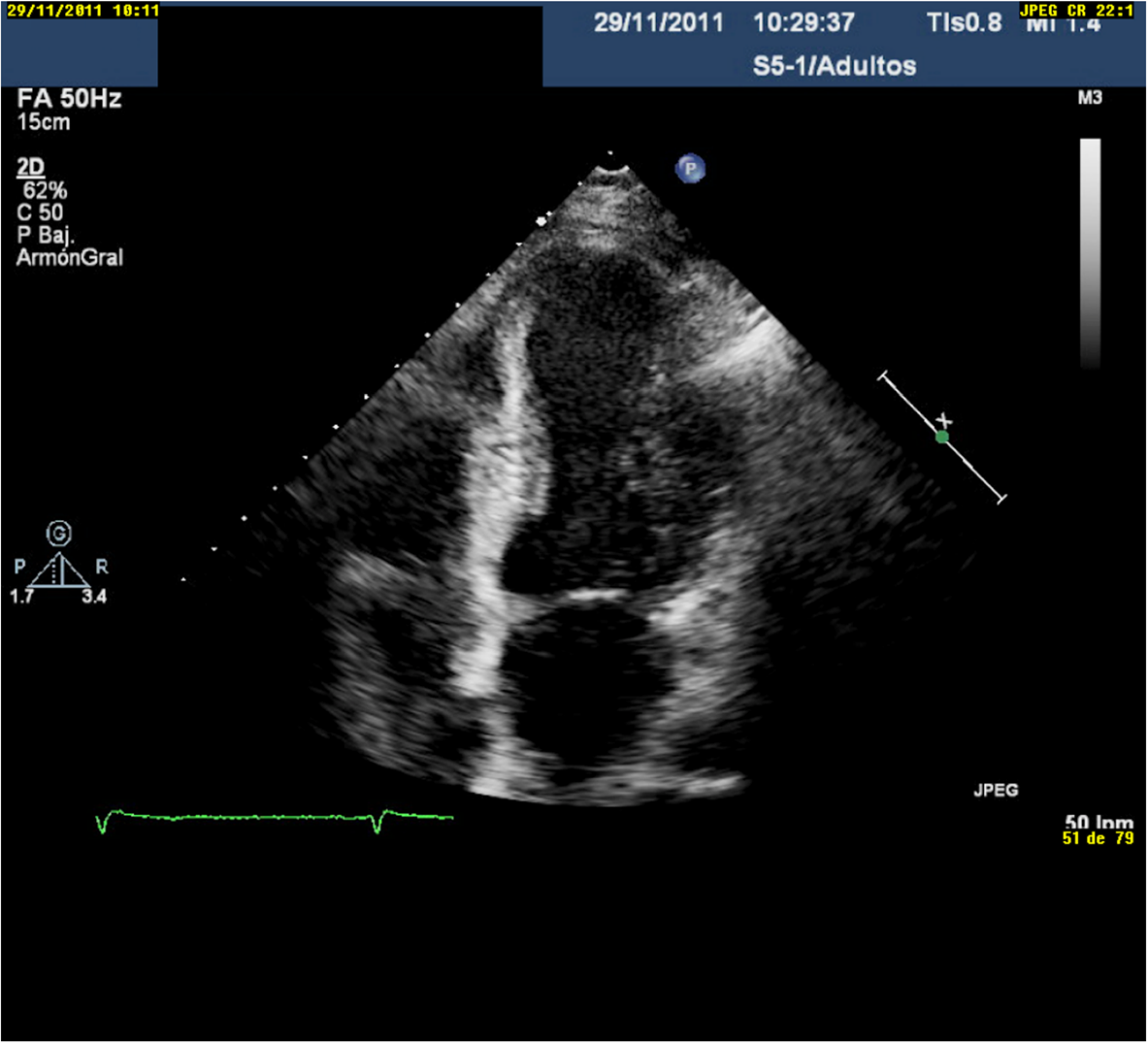
At 4 months after discharge, transthoracic echocardiography showed absence of left ventricular thrombus, persistence of apical-anterior and anterior akinesis, and resolution of the abnormal contractility in the area supplied by the revascularized right coronary artery.

**Figure 3:**
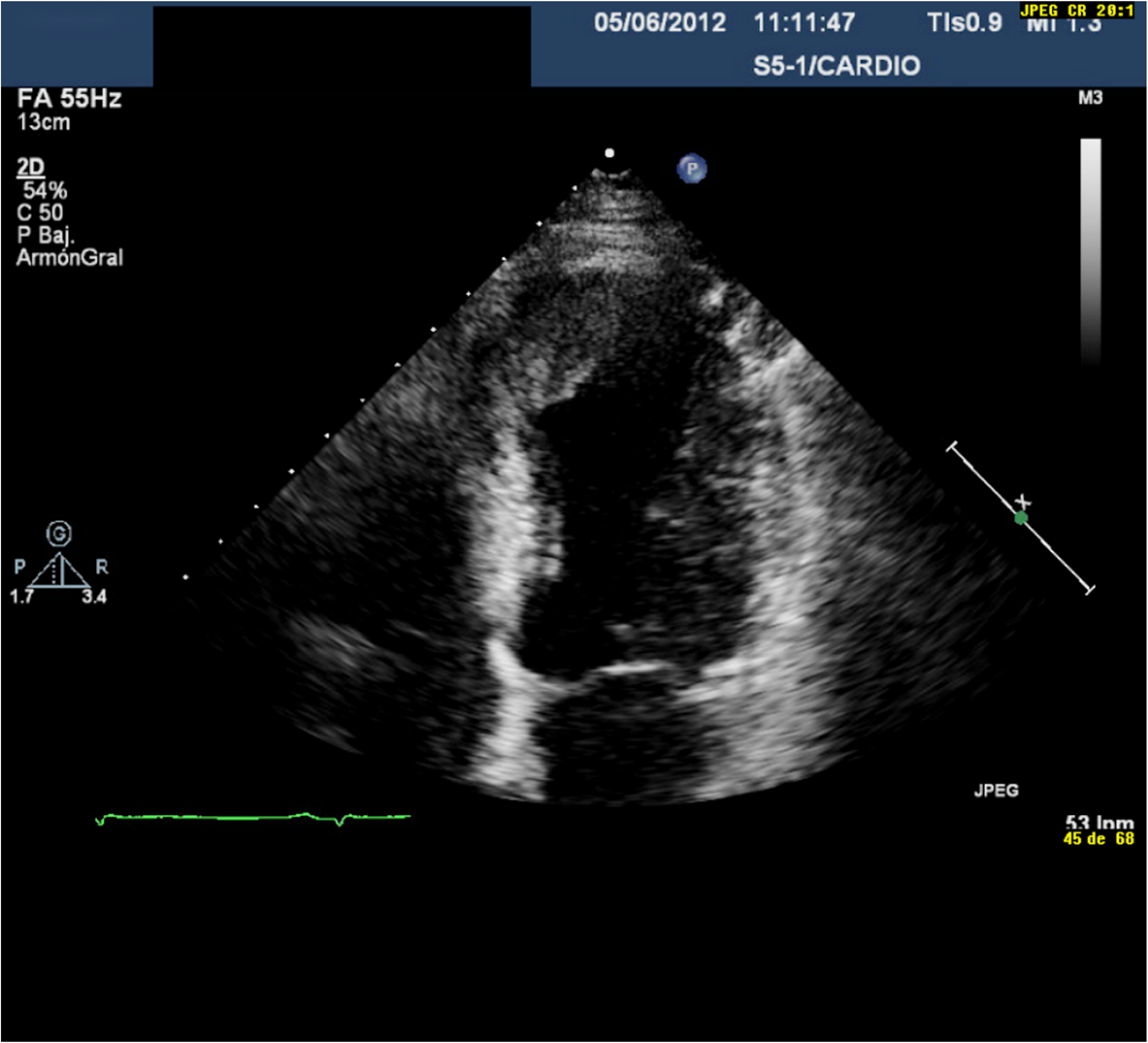
At 6 months after discharge, transthoracic echocardiography showed recurrence of apical thrombus, and the same abnormalities of left ventricular segmental contraction observed at 4 months after discharge.

A 66 year-old hypertensive man with a 10-year history of type II diabetes mellitus and a history of gastrointestinal bleeding was admitted with evolving inferior STEMI during 72 hours from the onset of symptoms. Electrocardiography (ECG) showed ST segment elevation in the inferior leads and Q waves in the inferior and anterior leads. The patient reported a similar episode of chest pain 1 month previously, for which he had not sought medical treatment. He had signs of low cardiac output during the first 2–3 days after admission, and was treated with amines. Evolving ECG changes suggested a subacute inferior myocardial infarction as well as an old anterior myocardial infarction. Coronary angiography on day 7 showed chronic occlusion of the mid-left anterior descending coronary artery and acute occlusion of the mid-right coronary artery (RCA), the latter being identified as responsible for the STEMI. Myocardial viability was assessed using cardiovascular magnetic resonance imaging, and percutaneous coronary intervention (PCI) was performed with placement of a drug-eluting stent (DES) in the RCA. The stent type was chosen because of the patient’s diabetes. Early after admission, TTE showed LV inferior hypokinesis, anterior and apical akinesis, and a large left intraventricular thrombus adherent to the septoapical and inferoapical endocardium (Figure 1, see Additional file 1: Video 1). The LV ejection fraction was 37%. The patient’s history and test results indicated a probable anterior STEMI 1 month before admission, and a subacute inferior STEMI. The D-dimer level was elevated at admission (785 μg/L) and normalized by the time of discharge (265 μg/L). Three weeks after admission, he was discharged on DAPT (clopidogrel and aspirin) and OAC (acenocoumarol). During the following 4 months, his international normalized ratio (INR) was maintained between 2.0 and 2.5. Ranitidine was prescribed because of the triple antithrombotic therapy and the history of gastrointestinal bleeding, but proton-pump inhibitors were not used. At 4 months after discharge, TTE showed absence of LV thrombus, persistence of LV apical-anterior and anterior akinesis, and resolution of the abnormal inferior wall contractility in the area supplied by the revascularized RCA (Figure 2, Additional file 2: Video 2). The LV ejection fraction was 47%. Considering the high risk of bleeding, OAC was stopped. Six months after discharge, TTE showed recurrence of apical thrombus, and the same abnormalities of LV segmental contraction observed at 4 months after discharge (Figure 3, Additional file 3: Video 3). At this point the D-dimer level was normal (251 μg/L). Laboratory tests were performed to rule out hypercoagulable state. His levels of fibrinogen, protein S, protein C, antithrombin III, Factor V Leiden, lupus antibody, and homocysteine were all normal. OAC was re-started on an indefinite basis, and DAPT was continued. One year after discharge, TTE showed absence of thrombus, and clopidogrel was stopped, but aspirin and OAC were continued. To date, the patient has not had any episodes of bleeding, and there has been no evidence of recurrent thrombus on regular TTE follow-up examinations.

## Discussion

Our decision to perform PCI with placement of a DES at the acute RCA lesion was guided by our patient’s diabetes and the evolving inferior myocardial infarction, after myocardial viability was confirmed on cardiovascular magnetic resonance imaging, in accordance with the current recommendations [1,7]. The reported chest pain 1 month before his admission and his ECG changes suggested an old anterior myocardial infarction. This was confirmed by changes in LV segmental contraction observed on TTE and invasive coronary angiography. The patient was discharged on DAPT, to be continued for at least 6 months, because of the type of stent used. OAC was also prescribed because of the LV apical thrombus, which was adjacent to the percutaneously revascularized ischemic endocardium. This therapeutic combination clearly increased the risk of bleeding, especially considering the patient’s age and history of hypertension and gastrointestinal bleeding [8]. The current consensus is that patients with mural thrombi should take OAC with vitamin K antagonist therapy for up to 6 months. However, this recommendation has not been revised in the era of stenting and DAPT. The optimal duration of triple antithrombotic therapy is unknown, and decisions regarding therapy should consider the relative risks of bleeding and stent thrombosis. If LV imaging after 3 months of therapy shows no evidence of thrombus, discontinuation of OAC earlier than 6 months can be considered, especially if there is recovery of apical wall motion [6]. Some authors have concluded that the duration of vitamin K antagonist therapy can be limited to 3 months in patients who have LV thrombus or are at risk for LV thrombus, such as those with antero-apical akinesis or dyskinesis, and that the duration of DAPT required may depend on the type of stent [3]. In our patient, follow-up TTE showed resolution of both the thrombus and the abnormal contractility of the apical-inferior and inferior LV supplied by the revascularized RCA. Our decision to stop OAC after 4 months was based on these findings, but DAPT was continued. However, the subsequent recurrence of thrombus in the same location suggests that thrombus formation was related to the non-revascularized apical-anterior and apical-septal myocardial segments, which were supplied by the left anterior descending coronary artery and had infarcted prior to admission.

There are some previous reports of cases similar to ours, in which LV thrombus recurred after discontinuation of OAC, causing cardioembolism years after STEMI [9,10]. When triple antithrombotic therapy is used, an international normalized ratio (INR) target range of 2.0 to 2.5 is reasonable [3], although prospective data for such treatment are lacking. Our patient had INR values between 2 and 3 in all follow-up tests, so we assume that the recurrent apical thrombus was not attributable to a failure of medical treatment. As he had risk factors for atherothrombosis, we did not initially suspect an underlying hypercoagulable state. As mentioned, D-dimer level was elevated at admission but had normalized at discharge, and at 4 months follow up. Although elevated D-dimer can indicate a hypercoagulable state, it is a non-specific marker that may also be elevated in various other conditions, including STEMI. After the recurrence of LV thrombus despite adequate triple antithrombotic therapy, laboratory tests for hypercoagulability were performed and all were normal. In our case we did not use GPIIb-IIIa antagonists, but the additive effects of GPIIb-IIIa antagonists and prasugrel’s active metabolite on platelet activation, aggregation, and procoagulant activities have been reported in STEMI patients [11]. Some authors have described prothrombotic genetic polymorphisms, such as PIA2, which is a determinant of ischemic stroke in a selected high-risk hypertensive population [12], or the single-nucleotide polymorphism of human CaMKIV gene (CaMK4), which plays a pivotal role in blood pressure regulation through the control of endothelial nitric oxide synthase (eNOS) activity. Impairment of CaMK-mediated activation of eNOS, as in CaMK4 gene deletion, induces hypertension, [13]. We were unable to test for genetic polymorphisms related to thrombosis, as described by other authors [12,13], as such testing is not available in our setting, and this is one of the principal limitations in the management of our patient.

When stent placement is required in a patient who needs subsequent long-term OAC, a bare-metal stent is generally preferred over a DES [2]. In patients without diabetes, small vessels, or large lesions requiring OAC because of atrial fibrillation or LV thrombus, the benefit of using a DES to prevent restenosis may be outweighed by the increased risk of bleeding with long-term triple antithrombotic therapy [14]. A bare-metal stent was not used in our case because the LV thrombus was detected after PCI.

The ACC/AHA guidelines [3] recommend 12 months of DAPT, but some preliminary data indicate that the rate of stent thrombosis may be very low when DAPT is discontinued at 6 months or maybe even 3 months after placement of a second-generation DES, such as an everolimus- or zotarolimus-eluting stent [15,16]. The randomized Efficacy of Xience/Promus versus Cypher to Reduce Late Loss in Stent (EXCELLENT) trial compared 6 months versus 12 months of DAPT, and everolimus-eluting versus sirolimus-eluting stents. The trial found that first-generation stents appear to have a higher rate of late stent thrombosis than second-generation stents, and second-generation stents are therefore preferred in patients who need a DES and subsequent triple antithrombotic therapy [17].

The recent randomized controlled What is the Optimal antiplatElet and anticoagulant therapy in patients with oral anticoagulation and coronary StenTing (WOEST) trial [18] found that among patients who underwent PCI with subsequent OAC, treatment with clopidogrel and OAC was associated with a significantly lower risk of bleeding than treatment with aspirin, clopidogrel, and OAC. The sample size was small, but the investigators saw no evidence of increased risk of thrombotic events by withholding aspirin. This suggests a possible alternative therapeutic approach for patients such as ours, using only OAC and clopidogrel. Physicians should be aware that genetic susceptibility to hypercoagulable states may favor the appearance or persistence of thrombus, related to mechanisms independent of the traditional cardiovascular risk factors. The need to investigate genetic polymorphisms related to thrombosis should be assessed on an individual basis.

## Conclusion

The present case illustrates the need for repeat TTE at least 3 months after the withdrawal of OAC in STEMI patients, as recommended by the guidelines. It is important to confirm the resolution of any LV thrombus and associated wall motion abnormalities. When there is occlusion of two or more coronary arteries supplying the apical area, it is not easy to determine which occlusion is related to thrombus formation. In patients undergoing primary PCI who require subsequent OAC, avoidance of a DES is strongly preferred. However, the benefits of a DES over a bare-metal stent may outweigh the risks in some cases. When a DES is deemed necessary, a second-generation stent is preferred if the patient requires subsequent triple antithrombotic therapy. An alternative regimen of OAC and clopidogrel, without aspirin, may be considered in such cases. In patients with recurrent intraventricular thrombus unexplained by inadequate antithrombotic therapy, genetic polymorphisms related to thrombosis should be investigated.

## Consent

Written informed consent was obtained from the patient for publication of this case report and any accompanying images. A copy of the written consent is available for review by the Editor-in-Chief of this journal.

## Competing interests

The authors declare that they have no competing interests.

## Authors’ contributions

JL analyzed and interpreted the patient’s data and wrote the paper. BM performed the TTE. AR and MMI were major contributors in writing the manuscript. AS and IL performed the PCI. All authors read and approved the final manuscript.

## Authors’ information

All authors are staff members of the Department of Cardiology, University Hospital of the Canary Islands, La Laguna, Tenerife, 38320, Spain.

## Supplementary Material

Additional files 1: Video 1 Early after admission, transthoracic echocardiography showed left ventricular inferior hypokinesis, anterior and apical akinesis, and a large intraventricular thrombus adhered to the septoapical and inferoapical endocardium. Click here for file

Additional files 2: Video 2 At 4 months after discharge, transthoracic echocardiography showed the absence of left ventricular thrombus, the persistence of apical-anterior and anterior akinesis, and recovery of impaired revascularized right coronary artery -dependent segmentary contractility. Click here for file

Additional files 3: Video 3 At 6 months after discharge, transthoracic echocardiography again showed the presence of an apical thrombus, as well as the same alterations of left ventricular segmentary contractility observed on transthoracic echocardiography at 4 months after discharge. Click here for file
